# cIMPACT‐NOW update 10: Recommendations for defining new types for central nervous system tumor classification

**DOI:** 10.1111/bpa.70018

**Published:** 2025-05-26

**Authors:** Cynthia Hawkins, Kenneth Aldape, David Capper, Andreas von Deimling, Caterina Giannini, Mark R. Gilbert, Thomas S. Jacques, David Jones, Takashi Komori, David N. Louis, Sabine Mueller, MacLean Nasrallah, Brent A. Orr, Arie Perry, Stefan M. Pfister, Felix Sahm, Chitra Sarkar, Matija Snuderl, David Solomon, Pascale Varlet, Pieter Wesseling, Guido Reifenberger

**Affiliations:** ^1^ Department of Paediatric Laboratory Medicine, The Hospital for Sick Children University of Toronto Toronto Ontario Canada; ^2^ Laboratory of Pathology, Center for Cancer Research National Cancer Institute Bethesda Maryland USA; ^3^ Department of Neuropathology Charité—Universitätsmedizin Berlin, Corporate Member of Freie Universität Berlin and Humboldt‐Universität zu Berlin Berlin Germany; ^4^ German Cancer Consortium (DKTK), Partner Site Berlin German Cancer Research Center (DKFZ) Heidelberg Germany; ^5^ Department of Neuropathology University Hospital Heidelberg Heidelberg Germany; ^6^ Clinical Cooperation Unit Neuropathology, German Consortium for Translational Cancer Research (DKTK) Deutsches Krebsforschungszentrum (DKFZ) Heidelberg Germany; ^7^ Department of Pathology Mayo Clinic Rochester Minnesota USA; ^8^ Department of Biomedical and Neuromotor Sciences (DIBINEM) Alma Mater Studiorum Bologna Italy; ^9^ Neuro‐Oncology Branch, Center for Cancer Research National Cancer Institute Bethesda Maryland USA; ^10^ UCL GOS Institute of Child Health and Department of Histopathology Great Ormond Street Hospital for Children London UK; ^11^ Division of Pediatric Glioma Research German Cancer Research Center (DKFZ) and German Cancer Consortium (DKTK) Heidelberg Germany; ^12^ Department of Laboratory Medicine and Pathology (Neuropathology) Tokyo Metropolitan Neurological Hospital Tokyo Japan; ^13^ Department of Pathology, Massachusetts General Hospital Brigham and Women's Hospital, and Harvard Medical School Boston Massachusetts USA; ^14^ Department of Neurology, Neurosurgery, and Pediatrics University of California San Francisco San Francisco California USA; ^15^ Department of Pediatric University of Zurich Zurich Switzerland; ^16^ Division of Neuropathology, Department of Pathology and Laboratory Medicine Perelman School of Medicine at the University of Pennsylvania Philadelphia Pennsylvania USA; ^17^ Department of Pathology St. Jude Children's Research Hospital Memphis Tennessee USA; ^18^ Department of Pathology University of California San Francisco San Francisco California USA; ^19^ Hopp Children's Cancer Center Heidelberg (KiTZ), Division of Pediatric Neurooncology German Cancer Research Center (DKFZ) and German Cancer Consortium (DKTK) Heidelberg Germany; ^20^ Department of Pediatric Hematology and Oncology Heidelberg University Hospital and National Center for Tumor Diseases (NCT) Heidelberg Germany; ^21^ Department of Pathology All India Institute of Medical Sciences New Delhi India; ^22^ Department of Pathology New York University Langone Health and Grossman School of Medicine New York New York USA; ^23^ Department of Pathology Stanford University School of Medicine Stanford California USA; ^24^ Department of Neuropathology GHU Paris—Psychiatry and Neuroscience, Sainte‐Anne Hospital Paris France; ^25^ Department of Pathology Amsterdam University Medical Centers/VU University Amsterdam The Netherlands; ^26^ Laboratory for Childhood Cancer Pathology Princess Máxima Center for Pediatric Oncology Utrecht The Netherlands; ^27^ Department of Neuropathology Heinrich Heine University Düsseldorf Düsseldorf Germany

Classification systems serve to group and organize data according to common relations or affinities so that they may be compared with other data. The classification system used will depend on what the classes are intended to cluster and what data are available to define these classes. Thus, the history and evolution of classifications trace the development of methods used to organize, categorize, and systematize knowledge, organisms, and objects based on shared characteristics. These systems have evolved over centuries, influenced by cultural, scientific, and technological advancements.

Tumor classifications have similarly evolved alongside advances in medicine, biology, and technology. These systems aim to categorize neoplasms based on various characteristics to improve diagnosis, prognostication, and treatment decisions. The earliest classifications relied primarily on clinical presentation, location, and macroscopic appearance of the cancer. Advances in microscopy and development of tissue staining techniques during the 19th century allowed pathologists to examine the cellular structure of tumors, as exemplified by the work of Virchow [1] who contributed significantly to the understanding of cancer as a disease originating from abnormal cells within the tissue. This era saw the introduction of histological classification systems, in which cancers were categorized based on their presumed tissue and/or cell of origin and the normal cells they resembled. In parallel, the concepts of benign versus malignant tumors were more clearly defined.

For central nervous system (CNS) tumors particularly gliomas, the early 20th century brought the first broadly recognized classification, published in 1926 by Bailey and Cushing [2]. This approach was based on a detailed study of a large series of brain tumors coupled with medical records of patients that had been followed from presentation to death; the goal was to provide better prognostic information and treatment planning, thus cementing clinical utility as a major endpoint of classification. In the mid‐20th century, efforts were undertaken to establish cancer classifications that could be used around the world. The initial World Health Organization (WHO) histologic classification manuals provided guidelines for categorizing tumors by their microscopic appearance and provided a framework for identifying cancer subtypes within specific organs [3].

The first edition of the WHO classification of CNS tumors was published in 1979 and included a grading system to distinguish tumors with presumed similar histogenesis (e.g., astrocytic) with different degrees of aggressiveness (e.g., pilocytic astrocytoma versus glioblastoma multiforme) [4]. This classification followed WHO Expert Committee on Health Statistics recommendations that specified three necessary elements of a classification: anatomic site, histologic tumor type, and grade as an indicator of the degree of malignancy [5]. For CNS tumors, the principles upon which grading would be based were (and in part remain) controversial. Zülch proposed five grades of clinical malignancy ranging from 0 to IV: grade 0 referred to extra‐parenchymal lesions amenable to surgical cure; grade I was considered benign but less reliably cured; grades II to IV ranged from borderline malignant to highly malignant and were usually lethal with different lengths of survival based on natural disease course (3–5 years, 1–3 years, and 0.5–1 year, respectively) [6]. Although not used in precisely this manner in more recent WHO classifications, this formalized the concept of “clinical” malignancy, rather than pure histologic malignancy, into subsequent WHO classifications for CNS tumors. This has, however, remained a somewhat difficult concept to implement with changing treatment paradigms and improved outcomes for many CNS tumors over time. Tumor grading, based on current outcomes versus “natural history” (defined as the potential clinical course of the tumor if left untreated) continues to be debated. Both systems have their inherent problems—the former would require potentially frequent grade changes with changing treatment paradigms, and even different grades for the same tumor depending on available treatments where the patient is diagnosed. The latter may lead to confusion when there is a significant gap between “natural history” and established clinical outcomes given current therapy (e.g., WNT‐activated medulloblastoma is still considered CNS WHO grade IV despite over 90% long term survival with current treatments).

The second and third edition of the WHO classification of CNS tumors (published in 1993 and 2000) evolved as clinical and biological knowledge increased [7, 8]. The 4th edition of the WHO classification of CNS tumors (2007) was significantly influenced by the now widespread use of immunohistochemistry to more accurately identify cell types and physiologically relevant cellular features such as proliferation [9]. This began an era of increasing classification complexity, coinciding with even more rapid technological developments. In the 2007 WHO classification, some groundwork was laid out regarding minimum criteria to be met for recognition as a distinct tumor type (albeit predating the use of molecular testing). These were: two or more reports from different institutions describing the tumor type, as well as distinct morphology, location, age distribution, and biologic behavior. Notably, the concept of histologic variants (now subtypes) and patterns was recognized: subtypes being recognizable histologically and having some relevance for clinical outcome but still part of a tumor type; and patterns recognizable histologically and thus important to note, but without distinct clinical significance. These concepts may also be applicable to molecular data.

The subsequent advent of high‐throughput genomic technologies over the past two decades, such as next‐generation sequencing, further transformed cancer classification. Large‐scale projects like The Cancer Genome Atlas (TCGA; https://www.cancer.gov/ccg/research/genome-sequencing/tcga) and the International Cancer Genome Consortium (ICGC; now ICGC‐ARGO https://www.icgc-argo.org/) mapped the genetic alterations in many cancers, leading to the development of classifications based on molecular signatures in addition to histology and immunohistochemistry. Incorporation of molecular features into tumor classification was first tackled by the neuro‐oncology community with the publication of the International Society of Neuropathology—Haarlem consensus guidelines for CNS tumor classification and grading in 2014 [10] and subsequently incorporated into the revised 4th edition of the WHO classification [11, 12]. This resulted in a number of new tumor types being introduced into the WHO classification of CNS tumors as well as a number of types lacking sufficient published evidence to make a decision (so‐called *sub judice*). However, the specific criteria needed to meet a new tumor type definition were not explicitly laid out.

With a growing number of advanced technologies that can be applied to classification and used to extend scientific knowledge, each classification update tends to become more complex. With the increasing use of transcriptomic, and in particular epigenetic (in this context, DNA methylation) profiling, tumor classification is becoming much more granular, as seen in the 5th edition (2021) WHO CNS tumor classification [13, 14]: there are more tumor types and more technologies recommended to diagnose those types. Nonetheless, while there are detectable molecular differences among these tumor types, sometimes very meaningful (e.g., WNT‐activated versus group 3 medulloblastoma), the differences may not always translate into changes in clinical behavior or therapeutic approaches (e.g., classical versus mesenchymal IDH‐wildtype glioblastoma). The situation thus poses the question: how does one most meaningfully define a tumor type? Further, what constitutes a new tumor type rather than a prognostic/grading marker in an existing type? These are not new questions, but rather ones that come up whenever new technologies yield a new stratum of data about a group of neoplasms.

Tumors are grouped based on commonalities (e.g., a clinical, histological or molecular feature); with increased molecular and clinical study, differences among members of a group of tumors will appear. The question is when do relatively minor distinctions that may be biologically or clinically important now or in the future warrant greater precision in designating tumor types. This is the longstanding debate between “lumpers” and “splitters” in the fields of pathology and oncology. The literature has a clear splitter bias as it is easier to publish findings demonstrating that a method of sub‐classification is associated with statistically significant differences; thus, most research articles over the last decade suggest the validity of further diagnostic sub‐classification distinctions. In contrast, clinical and treatment guidelines tend to lump together tumors that are molecularly distinct but do not currently have meaningfully different outcomes or treatment approaches. For example, post‐surgical treatment of IDH‐wildtype glioblastomas is mostly guided by *MGMT* promoter methylation status but not by histological or DNA methylation subtype.

Importantly, there are no clear rules guiding the incorporation of these distinctions into a classification. For example, when is a tumor sufficiently distinct from its near neighbors to warrant being considered a separate disorder, and when is the heterogeneity within a particular tumor type sufficient to warrant subdividing the group into more homogeneous subgroups (i.e., subtyping)? Of note, in reality there is no “ground truth” to be found in classifying tumors using increasing levels of technology. On the other hand, most would agree that ideally (1) changing concepts of tumor types and their recognition as distinct entities should be of prognostic and predictive significance, and (2) the complexity of a classification should not outstrip its clinical utility. Still, molecular distinctions within tumor types, if not clinically relevant now, could eventually be found to be important. This has been key in retrospective assessment of clinical datasets, as illustrated by older clinical trial cohorts for “primitive neuroectodermal tumors” (PNETs) which, with the benefit of modern molecular techniques, were eventually shown to encompass multiple distinct tumor types, including high‐grade gliomas, with divergent biologic implications.

With these challenges in mind, especially considering novel advanced technologies such as DNA methylation profiling, the Consortium to Inform Molecular and Practical Approaches to CNS Tumor Taxonomy (cIMPACT‐NOW) assembled a group of experienced neuropathologists and clinical neuro‐oncologists to provide recommendations regarding the definition of potential new tumor types for inclusion in the CNS tumor classification. While not intended to be applied to existing tumor types, the criteria may also help us to reorganize the existing taxonomy of CNS tumors in a meaningful way.

Two virtual conferences and one in‐person meeting (as well as numerous email correspondences) were held over approximately 12 months with a final consensus meeting in June 2024. There was a broad agreement that clearer criteria were required for defining new tumor types and that molecular features were important but not necessarily sufficient in isolation. The debates centered around grounding the entity definition in molecular versus clinical features and how much weight each of these should bear, how much to emphasize morphology, the possibility of tiered evidence, and what the burden of proof should be to show that a group of tumors represents a *distinct* type. With the understanding that any tumor types meeting these criteria would still need formal approval by the WHO committee, consensus recommendations are outlined below.

**A new CNS tumor type must be defined by:**

1. A clinical phenotype including typical prognosis/expected response to treatment, preferential age range, preferential location, and typical imaging features (grading within a tumor type may occur to reflect tumors with similar initiating events which typically progress over time)
**AND at least two among the following three criteria** (with fulfillment of all criteria being desirable but not always possible):

2 A unique clustering pattern based on large‐scale profiling (DNA methylation and/or RNA or protein expression profiles or multi‐omic data sets) compared to a sufficiently large reference cohort representing currently established entities; note that methylation or gene expression clusters driven solely by location‐dependent signatures do not qualify as a criterion for a distinct tumor type
3 An associated molecular profile comprising either a single or a combination of two or more genetic driver alterations (including single nucleotide variants, copy number variants, gene fusions, or other structural DNA alterations; a range of alterations may be found in the same tumor type if converging on the same oncogenic pathway[s])*
4 Associated microscopic (including immunohistochemical) features that are typical of, or at least closely linked to, the tumor type
Further (formal) prerequisites for a cIMPACT‐NOW recommendation as a (provisional**) new CNS tumor type are:

5 At least two independent publications reporting on the characteristic molecular, histological, and clinical features in well‐characterized patient cohorts of preferentially >10 patients each. Individual case reports are not sufficient, but multiple high‐quality case reports or small series could be considered for provisional designations in the case of very rare tumor types
**AND**

6 Approval by the relevant cIMPACT‐NOW Working Committee and by the cIMPACT‐NOW Steering Committee for recommendation as a new CNS tumor type to the WHO committee

*Genetic features relevant to cancer predisposition or targeted therapy potentially available to the patient should be reported in a layered diagnosis regardless of type/subtype.

**Will likely become a fully recognized type in a future classification but currently awaits further published characterizations.

While an attempt was made to be as precise as possible with these criteria, it should be noted that some of the definitions remain “soft,” namely what is meant by “preferential,” “associated,” or “typical/expected.” This may be seen as problematic, but it also allows for some flexibility in applying those parts of the definition and the reality of outliers in any working classification. Further, the criteria allow a CNS tumor type to be defined as such without the requirement of microscopic/histologic similarity among cases. While we expect this to be a rare situation and recognize that this is a difficult situation to come to terms with for those trained with a strong emphasis on morphology, clear examples of this scenario indeed exist, and therefore need to be accounted for. For example, glioblastomas IDH‐wildtype are microscopically/ histologically not always high grade, do not always have a prototypic glial/astrocytic phenotype in all areas (e.g., areas with primitive neuronal component), and otherwise bona fide desmoplastic small round cell tumors do not always have a desmoplastic and small round cell phenotype.

The participants were then asked to “field test” the new tumor type definition by applying it to a series of possible new types as well as several established types. Thirteen potential new tumor types, three WHO 2021 provisional types, and two existing types were tested. Both existing types (central neurocytoma and dysembryoplastic neuroepithelial tumor) were felt to meet the new proposed criteria of tumor types. Of the three current WHO 2021 provisional types, intracranial mesenchymal tumor, FET‐CREB fusion‐positive met all the criteria for a tumor type, but diffuse glioneuronal tumor with oligodendroglioma‐like features and nuclear clusters (DGONC) and cribriform neuroepithelial tumor (CRINET) were still considered as provisional on the basis of the small number of published cases. Of the proposed new types, three (astroblastoma with *EWSR1*::*BEND2* fusion; oligosarcoma, IDH‐mutant and 1p/19q‐codeleted and diffuse high‐grade glioma, IDH‐ and H3‐wildtype, arising after prior therapeutic radiation) were felt to represent subtypes, patterns or prognostic features of existing types, two (ependymoma‐like neuroepithelial tumor with *PLAGL1* fusions and neuroepithelial tumor with *PATZ1* fusions) were considered provisional types (see definition above and analogous to two of the three WHO 2021 provisional types). The remaining eight potential new tumor types (gliomas with *EP300::BCOR* or *CREBBP::BCORL1* fusions; CNS embryonal tumor with *PLAGL1/2* amplification; high‐grade glioma with pleomorphic and pseudopapillary features [HPAP]; tectal glioma with KRAS mutation; dural angioleiomyoma with *GJA4* mutation; glioneuronal tumor with *ATRX* alteration, kinase fusion, and anaplastic features [GTAKA]; CNS embryonal tumor with *BRD4*::*LEUTX* fusion and/or *CIC*::*LEUTX* fusion; intracerebral gliofibroma/schwannoma with VGLL3 fusion) did not yet meet the criteria of a (provisional) new type, many because they represented single studies and/or had limited clinical correlates. Importantly, these criteria evaluations were based on the literature available as of July 2024 and are not official recommendations, which would need to await the next WHO classification, and which would be based on the updated literature available at that time. In the meantime, these could be reported based on their morphologic appearance and immunohistochemical presumed histogenesis (i.e., astrocytic/ependymal/embryonal/other) and the addition of NEC (not elsewhere classified) and the molecular feature(s) in a layered fashion. This should help guide management, particularly for glial versus embryonal tumors, and flag that these are not typical members of these tumor types.

The consensus was to recommend adoption of these criteria for consideration of new tumor types being incorporated into CNS tumor classifications. The criteria appear to work in balancing the practicalities of some lumping while recognizing the potential splitting that advanced technologies bring to classification. It should also be noted that the relative significance of morphology, molecular findings, clinical presentation, age, location, and resectability are still not fully elucidated for some tumor groups (e.g., some of the newer glioneuronal tumors); all likely play a role in prognosis, but not all can be incorporated readily into a pathology‐based classification. As such, the criteria laid out here will ideally lead to a stricter approach to improve type assignments and will hopefully provide a framework for potential lumping of some of these tumor types moving forward.

The criteria outlined are intended to apply to new tumor types, but many of the same criteria can be applied to determine subtype versus pattern. Subtypes require a clinical impact, while patterns lack a clinical impact but are a recognizable variation in morphologic or molecular findings that is important to be aware of for diagnosis. Where one draws the line between a new subtype of an existing type versus a new type is somewhat arbitrary, but currently, attempts to adhere to the original broadly lineage‐based classification approach. We acknowledge that this has not been strictly adhered to in the past, with subtypes introduced without clear clinical implication. Similarly, the practice has been to not recognize prognostic markers as different subtypes, but this has not been universally applied.

The group urged international collaboration to accumulate well‐annotated sets of cases with longer clinical follow‐up for rarer tumor types to determine whether they represent true new types or are rather subtypes, or patterns, of existing types. This is particularly important as many molecular pathology‐based manuscripts lack the high‐quality clinical outcome data required to fully appreciate the clinical significance of the molecular finding. We trust that these recommendations prove useful to the field even when the next new influential technology comes along.

## Data Availability

Data sharing is not applicable to this article as no new data were created or analyzed in this study.
